# Non-Targeted Metabolomics Combined with Chemometrics by UHPLC–Orbitrap–HRMS and Antioxidant Activity of *Atractylodes chinensis* (DC.) Koidez. from Eight Origins

**DOI:** 10.3390/metabo13080888

**Published:** 2023-07-27

**Authors:** Xueyan Gao, Danyang Ma, Kaiyuan Li, Tianjiao Xing, Xiwu Liu, Lingfeng Peng, Dawei Chen, Zhihui Hao

**Affiliations:** 1 Chinese Veterinary Medicine Innovation Center, College of Veterinary Medicine, China Agricultural University, Beijing 100193, China; 2 Key Biology Laboratory of Chinese Veterinary Medicine, Ministry of Agriculture and Rural Affairs, Beijing 100193, China; 3 National Center of Technology Innovation for Medicinal Function of Food, National Food and Strategic Reserves Administration, Beijing 100193, China; 4 Qingdao Animal Husbandry Workstation, Qingdao 266100, China; 5 NHC Key Laboratory of Food Safety Risk Assessment, Chinese Academy of Medical Science Research Unit (No. 2019RU014), China National Center for Food Safety Risk Assessment, Beijing 100021, China

**Keywords:** *Atractylodes chinensis* (DC.) Koidez., origin, non-targeted metabolomics, chemometrics, antioxidant activity

## Abstract

*Atractylodes chinensis* (DC.) Koidez. (AC) is a type of Atractylodis Rhizoma that is widely used in China to treat diarrhea and arthritis, as well as a nutritional supplement. The objective of this study was to investigate and identify the phytochemicals in the aqueous extract of AC using an ultra-high-performance liquid chromatography (UHPLC)–Orbitrap–HRMS platform based on a non-targeted metabolomic approach. There were 76 compounds in the AC, the majority of which were phenylpropanoids (16) and terpenoids (15). The hierarchical clustering analysis (HCA) and principal component analysis (PCA) results revealed variations across eight AC samples and classified them into four groups. Using Pareto modeling, the orthogonal partial least squares-discriminant analysis (OPLS-DA) identified 11 distinct AC compounds. Furthermore, the antioxidant activity of eight AC samples was assessed using ABTS, DPPH, and OH· methods. The AC samples with concentrations ranging from 0 to 25 mg/mL had no toxic effects on A549 cells. They have a strong therapeutic potential against oxidation-related diseases, and further research on AC is warranted.

## 1. Introduction

*Atractylodes chinensis* (DC.) Koidez. (AC) is a type of Atractylodis Rhizoma, with a rich historical background and extensive usage in East Asia [1]. The distribution of AC is primarily found in the northern regions of China, including Hebei, Heilongjiang, Inner Mongolia, Liaoning, and other adjacent areas. The species AC, referred to as “Cangzhu” in the local context, is recognized for favorable treating conditions such as diarrhea [2], arthritis [3], hepatoprotection [4], asthma [5], and gastritis [6]. Atractylodis Rhizoma is a type of traditional Chinese medicine that has been included in the list of health foods published by the National Health Commission of the People’s Republic of China [7]. The usage of AC as a health food presents distinct benefits when compared to regular foods and pharmaceuticals. Compared to regular food, AC exhibits superior activity, particularly in enhancing spleen fortification and stomach harmonization [8], namely, alleviating functional dyspepsia symptoms, improving gastric emptying, and helping to improve the quality of life. The usage and dosage of AC are referred to in the specifications in the Pharmacopoeia of China. Additionally, the principle of compatibility in accordance with traditional Chinese medicine theory may be applied. As a result, the safety profile of AC may be superior. In Chinese history, AC used to be subjected to boiling water to produce various edible products such as tea, soup, and gruel. It also can be incorporated into biscuits to facilitate ease of consumption. Atractylodis Rhizoma food supplements are well-received in Japan, South Korea, Singapore, the United States, and other countries. Atractylodis Rhizoma is known to contain various constituents such as terpenoids, alkynes, glycosides, acyl sugar compounds, and other similar compounds [9]. These chemical components are identified by investigations of the alcoholic extract [10,11] and volatile components [12,13]. However, there is a lack of research on the aqueous extract.

Natural products comprise a complex chemical composition. The identification of the chemical constituents of natural products is advantageous for their subsequent application and advancement. Recent advancements in chemical composition analysis techniques have led to the exploration of more efficient and convenient operation methods, which have expanded the opportunities and possibilities for the analysis of natural products [14,15]. Non-targeted metabolomics is gaining popularity in the chemical components of natural products. Non-targeted screening aims to detect unknown compounds without any a priori criteria [16]. Ultra-high-performance liquid chromatography and high-resolution mass spectrometry (UHPLC-HRMS) can analyze chemical components using more efficient screening due to its selectivity, sensitivity, and high throughput. Quadrupole-Orbitrap can be used in both full-scan MS mode and MS/MS mode, allowing the identification of quasi-molecular ions and fragment ions [17], and is commonly employed in non-targeted analysis in the field of phytochemistry [18,19,20]. Orthogonal partial least squares-discriminant analysis (OPLS-DA) is commonly employed to evaluate dissimilarities among samples belonging to different groups. It can simplify the model and improve interpretability without reducing the predictive power [21], and it can analyze characteristic compounds.

The present study involved the collection of eight different AC origins. The geographical location may influence the chemical composition of AC. The aim of this study was to investigate compounds present in diverse AC samples through the employment of UHPLC–Orbitrap–HRMS. Subsequently, multivariate statistical analysis was employed to proficiently evaluate the characteristic compounds from diverse origins. The discrimination and classification of AC samples were clarified through the utilization of hierarchical cluster analysis (HCA), principal component analysis (PCA), and OPLS-DA techniques, with further analysis of their characteristic compounds. This study provides a method for screening the chemical components in the aqueous extract of AC that helps us identify the medicinal herbs of AC. The potential anti-oxidation properties of the AC sample were evaluated through the determination of its antioxidant activity, to enhance AC’s efficacy as an antioxidant.

## 2. Materials and Methods

### 2.1. Atractylodes chinensis (DC.) Koidez. Material

Eight kinds of AC samples were collected directly from different origins: Yanji (YJ), Baicheng (BC), Tieling (TL), Qikeshu (QKS), Baoshan (BS), Arun Banner (ARQ), Fujia Village Tianyi Town (FJ), and Harqin Banner (KLQ) in China. After taxonomic identification at the Institute of Medicinal Plant Development affiliated with the Chinese Academy of Medical Sciences by Yulin Lin, the mud was cleaned, the surface of the AC was sanded off, and then the samples were dried in the sun. After drying, the fibrous roots on its surface were knocked off and then crushed. The Chinese Pharmacopoeia (2020) thoroughly evaluated the quality of these samples, and the content of atractylodin in AC was in accordance with the standards.

### 2.2. Chemicals and Reagents

Standard references: Chlorogenic acid (CAS No.: 327-97-9), Neochlorogenic acid (CAS No.: 906-33-2), Isochlorogenic acid A (CAS No.: 2450-53-5), Isochlorogenic acid B (CAS No.: 14534-61-3), Atractylenolide I (CAS No.: 73069-13-3), Atractylenolide II (CAS No.: 73069-14-4), and Atractylenolide III (CAS No.: 73030-71-4) were purchased from Baoji Herbest Bio-Tech Co., Ltd. (Baoji, China). The purities of all the standard products are greater than 98%. The standard of Atractylodin (purity is 99.5%) was purchased from the National Institutes of Food and Drug Control (Beijing, China). Acetonitrile (HPLC grade) and formic acid (LC-MS grade) were purchased from Thermo Science, ammonium acetate (LC-MS grade) was purchased from J&K Scientific (Beijing, China), and ultrapure water was prepared from the Milli-Q system (Millipore Laboratory, Bedford, MA, USA). L (+)-Ascorbic acid (Vitamin C) was purchased from Sinopharm Chemical Reagent (Wuhan, China).

### 2.3. Preparation of the Aqueous Extract of Atractylodes chinensis (DC.) Koidez.

The *Atractylodes chinensis* (DC.) Koidez. was sieved to form AC powder (50 mesh). The 10 g AC powder was loaded into 250 mL round-bottom flask, 130 mL of purified water was added, and it was steeped for 1 hour at room temperature of about 25 °C and extracted by heating under reflux at 100 °C for 2 h. Then, the residue was filtered out using filter paper, and the filtrates were transferred into a 100 mL round-bottom flask and concentrated to 10 mL using a reduced-pressure rotary evaporator at 55 °C in order to obtain the aqueous extract of AC.

### 2.4. Preparation of AC Samples and Quality Control

In advance of injecting the aqueous extract sample into the LC-MS system, saccharide and protein precipitation was removed from all samples using acetonitrile, to prevent these impurities from interfering with the detection performance of the LC-MS system. Therefore, a portion (100 μL) of the aqueous extract of AC was mixed with acetonitrile (300 μL) and centrifuged for 15 min at 21,460× *g*, and the supernatant was filtered through a 0.22 μm pore-size nylon-66 filter membrane to further remove the impurities. The filtrate was analyzed on the UHPLC-Orbitrap-HRMS platform. A quality control (QC) sample was prepared by blending equal volumes of all the AC samples. By calculating the retention time of ions and the relative standard deviation (RSD) of peak area in QC samples, the stability and repeatability of the analytical method were examined.

### 2.5. UPLC-Orbitrap-HRMS Analysis

The experiments were carried out using a Thermo Fisher Scientific Ultra High-Performance Liquid Chromatography (UHPLC) system (Dionex UltiMate 3000) coupled with a high-resolution mass spectrometer (Q-Exactive, Thermo Fisher Scientific, Waltham, MA, USA). For instrument control and data collecting, the Xcalibur software (version 4.0) was employed.

The chromatographic separation was performed on an ACQUITY UPLC BEH C18 (100 mm × 2.1 mm, 1.7 μm, Waters, Milford, MA, USA) column at a flow rate of 0.2 mL/min and an injection volume of 3 µL. In the positive mode, ultrapure water-0.1% formic acid solution (Mobile Phase A) and acetonitrile-0.1% formic acid solution (Mobile Phase B) were used as mobile phases, while in the negative mode, ultrapure water-5 mM ammonium acetate solution (Mobile Phase A) and acetonitrile-5 mM ammonium acetate solution (Mobile Phase B) were used. The gradient elution time was 35 min, using the following multi-step gradient elution programs: 0–3 min (3–8% B), 3–12 min (8–20% B), 12–20 min (20–40% B), 20–25 min (40–85% B), 25–30 min (85% B), 30–30.2 min (85–3% B), and 30.2–35 min (3% B).

The MS data were acquired using electrospray ionization (ESI) in both negative and positive ionization modes, with spray voltage of 3 kV; sheath gas flow rate (N2, >95%) of 40; aux gas flow rate (N_2_, >95%) of 10; aux gas heater temperature of 320 °C (ESI+) and 300 °C (ESI−); and capillary temperature of 300 °C (ESI+) and 320 °C (ESI−). MS scanning mode: full MS scan spanned from *m*/*z* 100 to 1200, with the resolution of 70,000. MS/MS scanning mode: data-dependent MS2 scan (dd-MS2) with a resolution of 17,500 and normalized collision energy (NCE) was set to stepped (N) CE (15, 35, 55).

### 2.6. Data Processing and Identification

Compound Discoverer software (Thermo, version 3.2) was used to preprocess the raw data, which included peak detection, peak alignment, and peak filtering. Peak filtering works on the concept that the retention time range is 1.7~35 min, the mass range is *m*/*z* 100~1200, the mass error is ±5 ppm, and the minimum peak area is 100,000. The filtered molecular ion chromatographic peak and isotope peak match the possible molecular formula, and the measured spectrum of the secondary fragments is compared to the mzCloud database and the local Orbitrap traditional Chinese medicine library (OTCML). Based on reliable mass spectrum information, the software predicts the structure of the unknown compound. The screened compounds were scored with the mzLogic algorithm, and seven chemical compounds were validated using standards.

### 2.7. Chemometric Analysis

All of the data were normalized using total area sums in Excel 2019 before being analyzed. The peak lists of positive and negative ionization modes were further analyzed by HCA using R software (version 4.2.1) and PCA and OPLS-DA using SIMCA 14.1 (Umerics, Umeå, Sweden). The Euclidean distance was used as the distance measure in HCA. Univariate scaling was applied to the PCA model for the analyzed data, Pareto scaling was applied to OPLS-DA models, and automatic 7-fold cross-validation 200-time permutation tests were employed to evaluate the reliability of the OPLS-DA model. The quality of the established models was inspected by R^2^(cum) and Q^2^(cum), the values close to 1 indicate an excellent model [22]. The characteristic compounds were screened based on the criteria of the variable importance in the projection (VIP) value (VIP > 1) and correlation coefficient (p(corr), |p(corr)| ≥ 0.8) of the OPLS-DA model, together with the fold change (FC, |Log_2_(FC)| ≥ 1.5) [23].

### 2.8. Antioxidant Activities

Antioxidant activities were assayed in 5 mg/mL samples using 2,2′-azino-bis(3- ethylbenzothiazoline-6-sulfonate acid) (ABTS), 2,2-diphenyl-1-picrylhydrazyl (DPPH), and Hydroxyl (OH·) methods. At the same time, the antioxidant activities of different concentrations of Vitamin C (Vc; 0.025, 0.05, and 0.1 μg/mL) were also determined, which was frequently used as a positive control.

#### 2.8.1. Determination of Total Antioxidant Activities by ABTS

The total antioxidant activity was assayed using the ABTS method, as instructed by Beyotime Biotechnology [24]. In brief, the ABTS working mother liquor was made by mixing the two stock solutions in equal parts and storing it at room temperature in the dark for 24 h. Samples (10 μL) with 5 mg/mL were mixed with 200 μL of ABTS working solution and placed at room temperature for 6 min. Microplate readers (Infinite 200 pro, TECAN, Männedorf, Switzerland) were then used to measure the absorbance at 734 nm.

#### 2.8.2. Determination of Free Radical Scavenging Activity by DPPH

According to the instructions of the Nanjing Jiancheng Bioengineering Institute, the free radical scavenging activity was assayed by DPPH [24]. Briefly, the sample (400 μL) with a concentration of 5 mg/mL was mixed with 600 μL DPPH working solution and stored at room temperature in the dark for 30 min. The absorbance was then measured at 517 nm.

#### 2.8.3. Determination of the Hydroxyl Radical (OH)

The hydroxyl radical scavenging activity was assayed by the Fenton reaction according to the instruction of Nanjing Jiancheng Bioengineering Institute [25]. Briefly, the sample (200 μL) was mixed with substrate solution (200 μL) and reagent III (400 μL). The mixture was reacted at 37 °C for exactly 1 min, and then the chromogenic reagent (2 mL) was added immediately. After mixing, the mixture was placed at room temperature for 20 min. The absorbance was then measured at 550 nm.

### 2.9. Cytotoxicity Assays

Cytotoxicity was measured with a CCK-8 assay (MedChemExpress Monmouth Junction, NJ, USA). Briefly, A549 cells were plated at a concentration of 1 × 10^4^ cells/mL in 96-well plates and grown overnight in 100 µL Dulbecco’s Modified Eagle Medium (Gibco). Then, the cells were incubated with different concentrations of toxins at 37 °C for 24 h. Each dose gradient had three replicates. After which, 10% CCK-8 solution was added to each well and incubated for 2 h. The absorbance was at 450 nm.

### 2.10. Statistical Analysis

Statistical analysis was performed with SPSS version 21.0 program package (IBM, Armonk, NY, USA). The difference between two groups was tested by Student’s *t*-test. A *p*-value of <0.05 (*p* < 0.05) was considered for a significant difference (* *p* < 0.05, ** *p* < 0.01, *** *p* < 0.001).

## 3. Results and Discussion

### 3.1. Identification Results of Chemical Components of Atractylodes chinensis (DC.) Koidez.

The 76 phytochemicals were screened in AC samples using calculated molecular weight, *m*/*z* of parent ion and fragment ions, and then seven components (Chlorogenic acid, Neochlorogenic acid, Isochlorogenic acid A, Isochlorogenic acid B, Atractylenolide I, Atractylenolide II, and Atractylenolide III) were identified using commercially available standards based on the UHPLC–Orbitrap–HRMS platform with their retention time, accurate molecular mass (Table S1), and chemical structure (Figure S2). The residual components were identified using the non-targeted analysis strategy proposed in Section 2.6 of this study. In this study, phenylpropanoids (16) and terpenoids (15) were the main components in AC samples. The remaining compounds were eight phenols, five polyacetylenes, four alkaloids, four amino acids, four lignans, three flavonoids, three glycosides, three organic acids, two lactones, and nine miscellaneous. The literature reports the identification of approximately 35 compounds in Atractylodis Rhizoma, among which are Chlorogenic acid, 5-O-Feruloylquinic acid [26], (6E,12E)-Tetradecadiene-8,10-diyne-1,3-diol, 5-Hydroxymethylfurfural, Atractylenolide III [27], and Atractyloyne [28]. This discovery provides a chemical substance basis for the further exploitation and utilization of AC.

For example, the mass spectra of compound **2** analyzed in full-scan mode indicated that it was present mainly in a singly charged protonated form ([M+H]^+^, *m*/*z* 268.10333). MS2 fragmentation resulted in main fragments at *m*/*z* 136.06184 and 119.03561, originating from the neutral loss of C_5_H_8_O_4_ and NH_3_, respectively. Based on the mass spectrometric fragmentation patterns and literature reports [29], it is speculated that the compound is Adenosine (Figure S3a).

The mass spectra of compound **5** analyzed in full-scan mode indicated that it was present mainly in a singly charged protonated form ([M+H]^+^, *m*/*z* 152.05635). MS2 fragmentation resulted in main fragments at *m*/*z* 135.03018, 110.03527, and 109.05110, originating from the neutral loss of NH_3_, CH_2_N_2_, and CHON, respectively. According to its mass spectrum information and the information from the previous literature [30], it was identified as Guanine.

The mass spectra of compound **10** analyzed in full-scan mode indicated that it was present mainly in a singly charged protonated form ([M+H]^+^, *m*/*z* 166.08590). MS2 fragmentation resulted in main fragments at *m*/*z* 120.08102, 149.06026, and 103.05472, originating from the neutral loss of COOH, NH_3_, and COOH plus NH_3_, respectively. According to its mass spectrum information and the information from the previous literature [31], it was identified as L-Phenylalanine (Figure S3b). Based on the above fragmentation pattern, it is inferred that compounds **1**, **7**, and **16** are L-Tyrosine, L-Isoleucine, and D-Tryptophan, respectively.

The mass spectra of compound **24** analyzed in full-scan mode indicated that it was present mainly in a singly charged deprotonated form ([M-H]^−^, *m*/*z* 505.17175). MS2 fragmentation resulted in main fragments at *m*/*z* 487.16052, 475.15967, and 343.11899, originating from the neutral loss of H_2_O, CH_2_O, and C_6_H_10_O_5_, respectively. According to its mass spectrum information and the information from the previous literature [32], it was identified as 4,6,2′,4′-Tetramethoxychalcone 2′-beta-glucoside.

The mass spectra of compound **29** analyzed in full-scan mode indicated that it was present mainly in a singly charged protonated form ([M+H]^+^, *m*/*z* 193.04890). MS2 fragmentation resulted in main fragments at *m*/*z* 178.02605, 165.05510, 150.03107, and 133.02850, originating from the neutral loss of CH_3_, CO, CH_3_ plus CO, and CH_3_OH plus CO, respectively. According to its mass spectrum information and the information from the previous literature [33], it was identified as Scopoletin (Figure S3c).

### 3.2. Hierarchical Cluster Analysis (HCA) of AC Samples

The standardized normalized peak area was used as a variable in this study, and hierarchical clustering analysis (HCA) was used to classify AC samples by hierarchical decomposition. Meanwhile, a heatmap was used to represent activity and aggregation status more intuitively.

**Figure 1:**
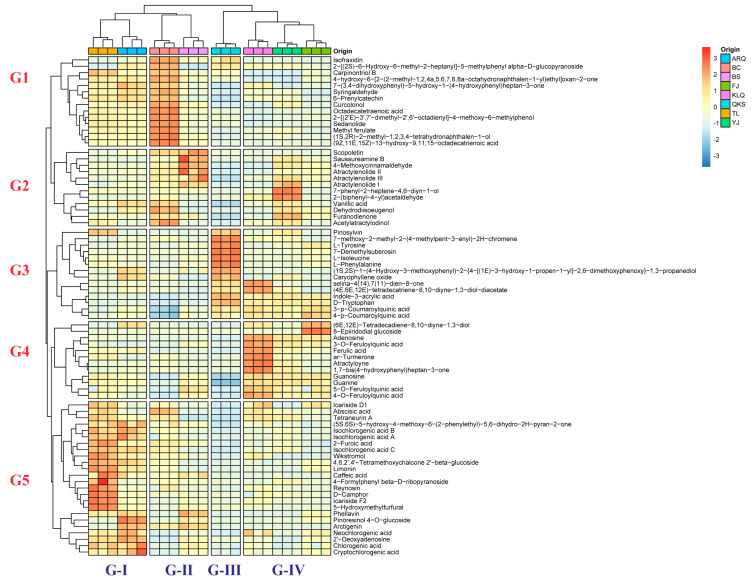
Heatmap of hierarchical cluster analysis of 76 compounds in aqueous extracts of AC from 8 different origins. The AC samples were classified into 4 groups: G-I, G-II, G-III, and G-IV. Seventy-six compounds were divided into 5 groups: G1, G2. G3, G4, and G5.

In the samples, all of the components were noticeably different. The heatmap was created to represent the differences between AC samples in an intuitive way. The AC samples were classified into four groups: Group-I (G-I), Group-II (G-II), Group-III (G-III), and Group-IV (G-IV). The AC samples in G-I are KLQ and TL, the AC samples in G-II are BS and FJ, the AC sample in G-III is QKS, and the AC samples in G-IV are ARQ, BC, and YJ. Simultaneously, 76 compounds were divided into five groups. The first group (G1) contains 14 compounds, including Isofraxidin, Carpinontol B, Syringaldehyde, 6-Prenylcatechin, Curcolonol, and others. The second group (G2) is composed of 12 compounds, including Scopoletin, Saussureamine B, Vanillic acid, 4-Methoxycinnamaldehyde, Atractylenolide I, and others. The third group (G3) contains 13 compounds, such as D-Tryptophan, 3-p-Coumaroylquinic acid, 4-p-Coumaroylquinic acid, Pinosylvin, L-Tyrosine, etc. The fourth group (G4) contains 12 compounds, such as 3-O-Feruloylquinic acid, 4-O-Feruloylquinic acid, 5-O-Feruloylquinic acid, Ferulic acid, Atractyloyne, etc. The fifth group (G5) contains the remaining 24 compounds. According to Figure 1, the proportion of G3 compounds is the highest in the aqueous extract of AC from G-III, while the proportion of compounds is low in the other four groups. The proportion of G5 compounds in the aqueous extract of AC from G-I is the highest, while the proportion of G4 compounds in G-IV is also relatively high. In comparison to other groups, the proportion of G1 and G2 compounds in G-II aqueous extract is relatively high.

### 3.3. Principal Component Analysis (PCA) of AC Samples

PCA is a multivariate method that is commonly used to summarize data variations, reveal differences between groups, and quantify the variability of samples within the same group [34]. It was applied in order to discriminate and analyze the compounds identified in the aqueous extract of AC from eight different origins, producing more intuitive classification results and the detection of abnormal samples.

**Figure 2:**
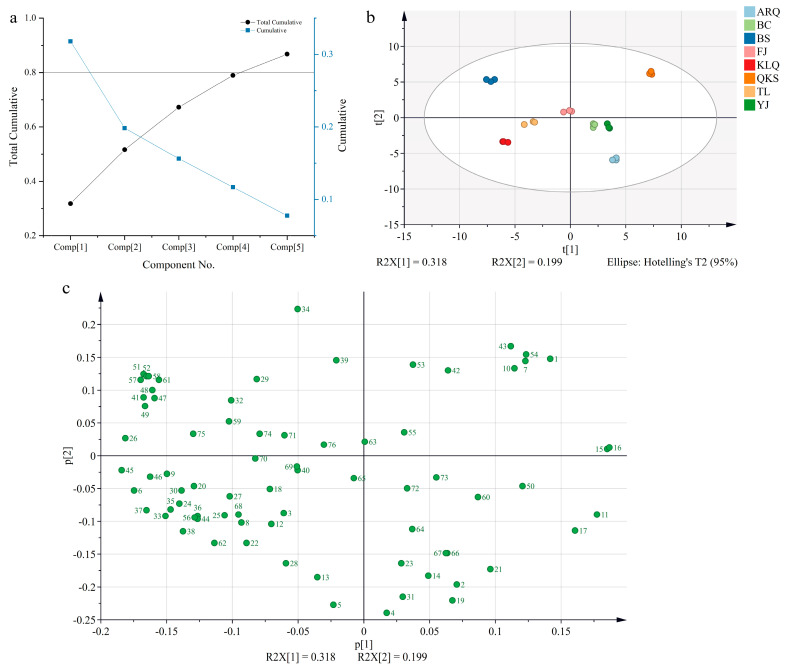
Variation map (**a**), score plots (**b**), and loading plots (**c**) were based on principal component analysis (PCA) of compounds in aqueous extract of AC from different regions.

To preserve as much of the original information as feasible, the principal component with the greatest variance of the explanatory variables was selected. The overall cumulative variance contribution rate exceeds 0.8 when the number of principal components reaches five. As a result, selecting the top five principal components can effectively retain the information from the original sample. In terms of the interpretable variance of each principal component (PC) (Figure 2a), PC1 > PC2 > PC3 > PC4 > PC5, where the variance contribution rates of PC1 and PC2 are 31.80% and 19.83%, respectively, and the cumulative variance contribution rate is 51.63%. The PCA score plots revealed that the chemical composition of the aqueous extract of AC samples from eight distinct origins varied (Figure 2b). The QKS and BS samples were mostly distributed in the first and second quadrants, respectively. AC samples from TL and KLQ were placed in the third quadrant, while samples from BS, YJ, and ARQ were placed in the fourth quadrant. Only the BC sample was shown in both the first and second quadrants.

The loading plots reveal the contribution value of each compound to sample categorization, indicating that the distribution of 76 compounds was somewhat distributed (Figure 2c). The further the compound is from the base point, the greater the contribution to the classification of all samples, such as D-Tryptophan (0.187, PC1), Indole-3-acrylic acid (0.185, PC1), 6-Prenylcatechin (−0.184, PC1), Isofraxidin (0.224, PC2), Guanosine (−0.240, PC2), Guanine (−0.228, PC2), 4-O-Feruloylquinic acid (−0.221, PC2), and Ferulic acid (−0.215, PC2).

### 3.4. Orthogonal Partial Least Squares-Discriminant Analysis (OPLS-DA) of AC Samples

The compounds were evaluated by OPLS-DA based on the results of HCA and PCA to further investigate the differences between the aqueous extracts of AC samples from diverse sources and identify potential characteristic compounds.

OPLS-DA is a method that combines supervised partial least squares-discriminant analysis (PLS-DA) and orthogonal signal filtering to accurately estimate differences between two or more groups and identify biomarkers [35]. It can extract the distinct information between groups more effectively than PCA. The AC samples were pre-classified in the SIMCA software, and the grouping is consistent with HCA results. There is a definite tendency of separation among the four groups after dividing the AC samples from eight generating regions into four categories (Figure S4). The R^2^X (cum), R^2^Y (cum), and Q^2^ (cum) values produced by the model are 0.751, 0.988, and 0.978 (Table 1), respectively, which fulfills classification predictions. Therefore, the chemical compounds were evaluated on this basis in order to identify the characteristic chemical compounds.

**Figure 3:**
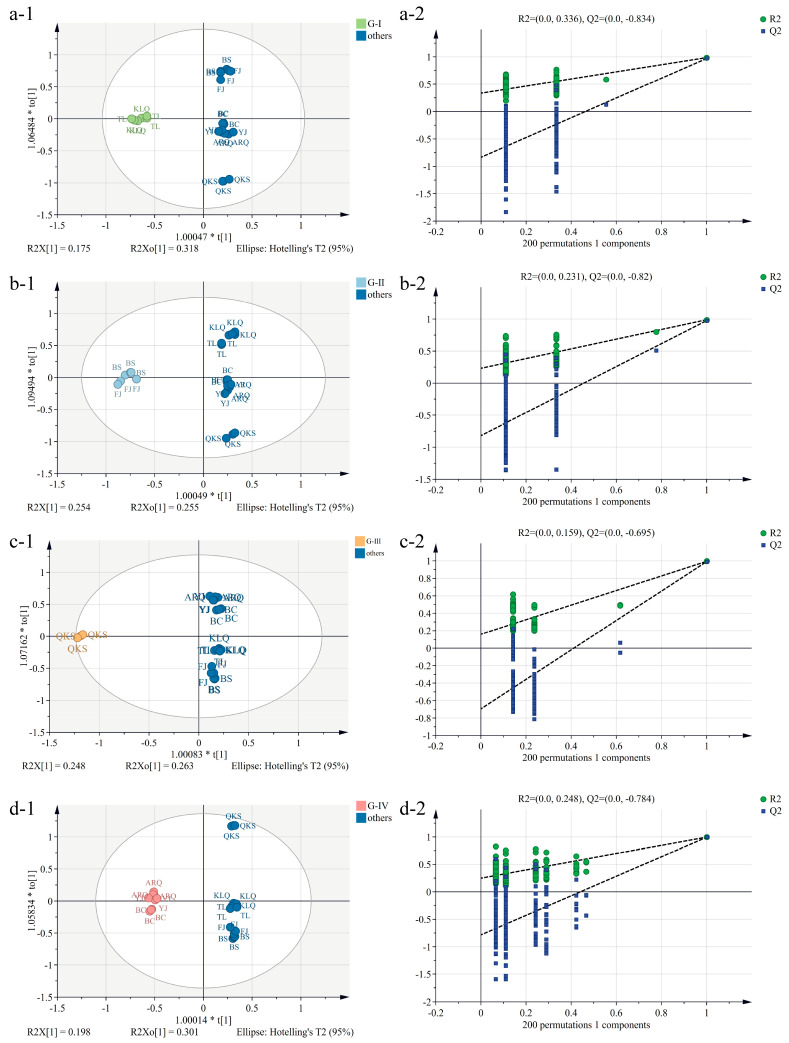
OPLS-DA score plots (1) and 200-time permutation test plots (2) of the aqueous extracts of AC samples from different origins in M2 (**a**), M3 (**b**), M4 (**c**), and M5 (**d**).

**Figure 4:**
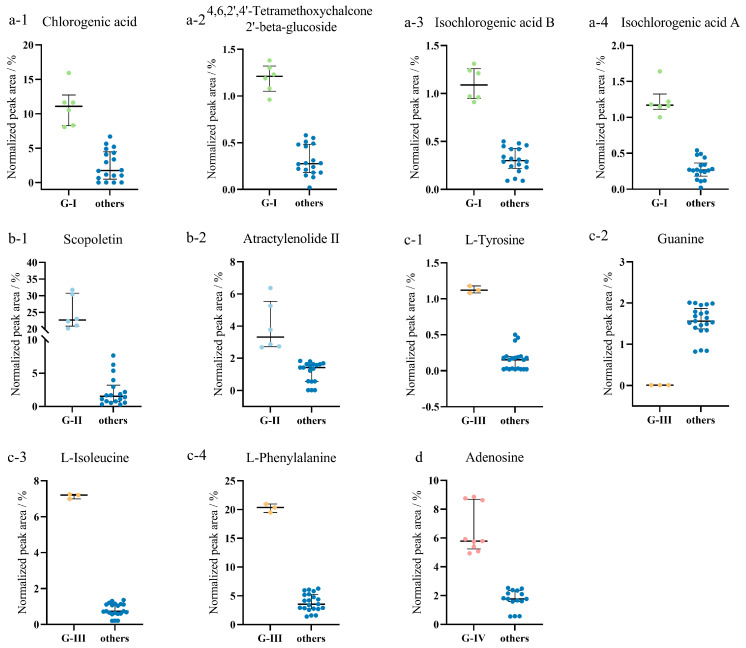
Normalized peak area (%) of the 11 characteristic compounds of (**a**) G-I, (**b**) G-II, (**c**) G-III, and (**d**) G-IV.

Using OPLS-DA to compare and analyze the results of different groups and all other origins, the values of R^2^X (cum), R^2^Y (cum), and Q^2^ (cum) in the four models and the number of variables with VIP > 1 were calculated (Table 1). A 200-iteration permutation test was then performed to avoid the model from being over-fit. In Figure 3, the R^2^ (cum) and Q^2^ (cum) values on the left are lower than the original point on the right, and the regression line of Q^2^ has a negative intercept, indicating that the model has favorable prediction capacity and no over-fitting, and thus potential biomarkers may be selected accordingly. Table S2 displays the VIP, p(corr), and Log_2_(FC) values of the obtained characteristic compounds. Eleven compounds from different origins were assessed as characteristic compounds of AC. Scopoletin and Atractylenolide II are the characteristic compounds of AC samples from FJ and BS. The AC characteristic compounds generated in KLQ and TL are Chlorogenic acid, Isochlorogenic acid A, 4,6,2′,4′-Tetramethoxychalcone 2′-beta-glucoside, and Isochlorogenic acid B. Adenosine is a characteristic compound for AC in ARQ, BC, and YJ. The compounds L-Phenylalanine, L-Isoleucine, Guanine, and L-Tyrosine are considered characteristic of QKS. However, Guanine was not detected in QKS, and L-Tyrosine was found to be absent in KLQ, ARQ, and BS. These compounds also differed significantly in the normalized peak area (Figure 4) and the HCA heatmap (Figure 1). Extra AC samples should be gathered in the future to demonstrate the result of the potential characteristic compounds.

### 3.5. Antioxidant Activity Analysis

The antioxidant activity evaluation of foods and biological samples is critical for maintaining the quality of functional foods and investigating the efficacy of dietary antioxidants on oxidative stress illnesses [36]. The antioxidant activity of several AR samples (5 mg/mL) was determined using OH·, DPPH, and ABTS radical scavenging. The results were compared to the antioxidant activity of Vc at various concentrations (0.025, 0.05, and 0.1 mg/mL).

**Figure 5:**
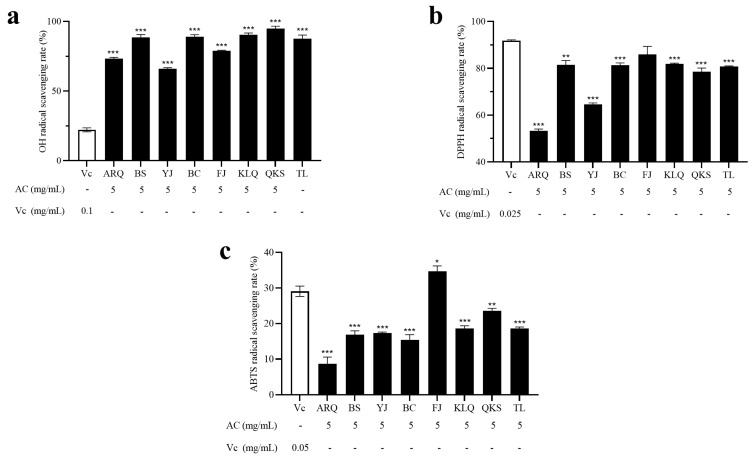
The antioxidant capacities of AC were determined using (**a**) OH· radical scavenging assay, (**b**) DPPH radical scavenging assay, and (**c**) ABTS radical scavenging assay. The data are mean values based on three replicates ± SD. For the significant difference compared to Vc, a *p*-value of <0.05 (*p* < 0.05) was used (* *p* < 0.05, ** *p* < 0.01, *** *p* < 0.001).

The Fenton reaction can generate OH· radicals, which react with the Griess reagent to form red substances. The amount of OH· radicals determines the color depth. The aqueous extract of AC has a scavenging ability of OH· radicals of more than 65% (Figure 5a). The scavenging capacity of the AC samples from ARQ, YJ, and FJ was 73.26%, 65.97%, and 78.91%, respectively. QKS has the highest OH· radical scavenging rate (94.80%).

DPPH is a stable free radical that becomes purple when dissolved in ethanol. DPPH can decolorize when it interacts with antioxidants, indicating the antioxidant’s ability to scavenge DPPH free radicals. The results revealed that the DPPH radical scavenging ability of the aqueous extract of AC from six origins exceeded 75%, and its scavenging ability was FJ > KLQ > BS > BC > TL > QKS (Figure 5b).

Under the action of appropriate oxidants, ABTS can be oxidized to green ABTS+. When antioxidants are introduced to the system, the production of ABTS+ is inhibited, and the degree of color development is linearly linked to the antioxidant capacity, which might indicate the total antioxidant capacity of the extract. The results showed that the ABTS radical scavenging capacity of FJ was the greatest (34.71%), followed by QKS (23.51%), while that of the other six producing locations was less than 20%, with ARQ (8.70%) being the lowest (Figure 5c).

Among the existing literature, this article stands out as the first work to assess the antioxidant potential of AC aqueous extract, revealing its impressive antioxidant potency. The same aqueous extract showed different scavenging capacities. In terms of OH· radical scavenging capacity, AC samples (5 mg/mL) outperformed Vc (0.1 mg/mL) by at least 40%. Nevertheless, in terms of DPPH radical and ABTS radical scavenging capacity, the AC samples and Vc (0.025 and 0.05 mg/mL, respectively) were comparable. The structure of a compound determines its ability to scavenge DPPH and ABTS radicals, and ABTS radical scavenging ability is more suitable for measuring dihydrochalcone- or flavanone-rich extracts, while the DPPH assay was mainly determined by the number of OH groups [37]. The ability of compounds with similar structures to scavenge DPPH radicals is related to the number and position of hydroxyl groups in their aromatic rings, but the results of ABTS free radical scavenging ability are different from those of DPPH radicals [38]. This may result in AC exhibiting higher DPPH radical scavenging ability and lower ABTS radical scavenging ability. The DPPH assay measures the ability of an antioxidant to donate hydrogen to a free radical with an ethanol system [39], while the OH assay measures were using a water system. OH assay may better reflect the antioxidant capacity of the aqueous extract itself. A study found that the antioxidant activity of some plants was positively correlated with their scopoletin content [40]. Atractylenolide II shows potential antioxidant properties and may block DNA damage and ROS-induced cell death [41]. A study on *Flos Lonicerae* extracts suggests that it had antioxidant activity, and chlorogenic acid was the main contributor to this activity [42]. In addition, some amino acids also have antioxidant properties. Tryptophan can efficiently clean free radicals, including reactive oxygen species and activated oxygen species [43]. Phenylalanine has been shown to increase the production of antioxidants, which can help protect against oxidative stress [44]. The in vitro antioxidant activity showed a potent free radical scavenging activity for furanodienone as evidenced by a low IC_50_ value for DPPH radicals (1.164 ± 0.58 mg/mL) [45]. Overall, these findings indicated that the aqueous AC samples have high anti-oxidant activity. Radical scavenging activities are very important to prevent the deleterious role of free radicals in different diseases, including cancer. Free radicals produced under environmental stimuli can directly or indirectly oxidize or damage DNA, proteins, and lipids, inducing gene mutations, protein denaturation, and lipid peroxidation, thereby leading to pathological processes [46]. The antioxidant capacity of AC endows it with the potential to resist the damage caused by oxidative stress to the body, which may have important applications in the future.

### 3.6. Cytotoxicity Analysis

**Figure 6:**
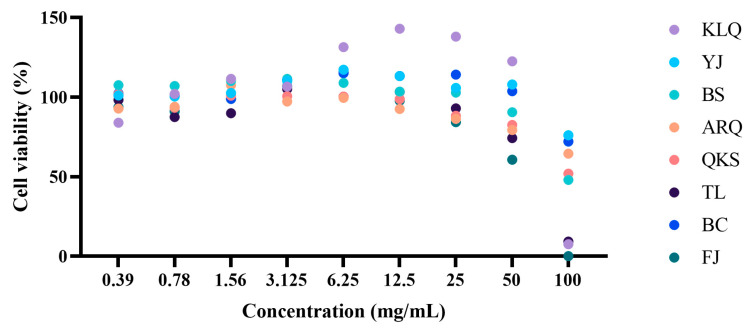
The cell viability (%) of AC was determined using CCK-8 assay. The data are mean values based on three replicates.

Considering the potential toxicity of AC [47], the cytotoxicity on A549 cells was tested. The results showed that the extracts with concentrations ranging from 0 to 25 mg/mL had no toxic effects on A549 cells, with cell viabilities higher than 80% (Figure 6). It indicates that AC aqueous extract is safe for A549 cells within this concentration range. At a concentration of 50 mg/mL, AC water extracts from FJ exhibited cytotoxicity; the cell viability was 60.66%. At a concentration of 100 mg/mL, as the concentrations of AC aqueous extract from KLQ, TL, and FJ increased to 100 mg/mL, no viable A549 cells were noticed, and the cell viability was 7.59%, 9.18%, and 0%, respectively. The toxicity of AC may be related to Atractyloside [48], and therefore, the presence of Atractyloside in the aqueous extract was rechecked. In this study, the response of Atractyloside was poor in the QC samples, which may be related to the LC-MS system.

## 4. Conclusions

The phytochemicals in the aqueous extract of *Atractylodes chinensis* (DC.) Koidez. from eight different origins were examined using UHPLC–Orbitrap–HRMS in this work. A total of 76 compounds were identified; the primary compounds were phenylpropanoids (16) and terpenoids (15), with the other compounds containing eight phenols, five polyacetylenes, four alkaloids, four amino acids, four lignans, three flavonoids, three glycosides, three organic acids, two lactones, and nine miscellaneous. The HCA and PCA methods were used to demonstrate the difference between AC samples from different origins. It was intuitively clear that the chemical composition in the aqueous extract of AC samples from eight distinct origins was discriminatory. To investigate the potential characteristic compounds, the OPLS-DA with a pairwise model was applied. Eleven compounds were discovered to be useful in distinguishing four groups of AC samples from various sources. Additionally, the antioxidant activity of AC was assessed by OH radical, DPPH, and ABTS scavenging methods, showing that the antioxidant capacity of aqueous extracts from different origins varied and was outstanding. The aqueous extract of AC with concentrations ranging from 0 to 25 mg/mL from different origins had no toxic effects on A549 cells. It is imperative to conduct comprehensive research on the aqueous extract of *Atractylodes chinensis* (DC.) Koidez in the future.

## Figures and Tables

**Table 1:** Parameters of OPLS-DA models based on different groups of AC.

Model	A	N	R^2^X (cum)	R^2^Y (cum)	Q^2^ (cum)	VIP > 1 Number
M1: Comparison between all groups	3 + 1 + 0	24	0.751	0.988	0.978	—
M2: G-I vs. others	1 + 3 + 0	24	0.724	0.985	0.968	22
M3: G-II vs. others	1 + 2 + 0	24	0.59	0.989	0.976	12
M4: G-III vs. others	1 + 1 + 0	24	0.51	0.995	0.992	13
M5: G-IV vs. others	1 + 2 + 0	24	0.649	0.997	0.994	15

## Data Availability

The data presented in this study are available on request from the corresponding author. Data is not publicly available due to privacy.

## Supplementary Materials

The following supporting information can be downloaded at https://www.mdpi.com/article/10.3390/metabo13080888/s1, Figure S1: The total ion chromatograms of the UHPLC-Orbitrap-HRMS analysis of *Atractylodes chinensis* (DC.) Koidez. samples, with a focus on the (a) negative ion and (b) positive ion, Figure S2: The chemical structure of 76 compounds identified in this study, Figure S3: Mass spectrometric fragmentation patterns of (a) Adenosine, (b) L-Phenylalanine, and (c) Scopoletin, Figure S4: OPLS-DA results of AC from 8 different regions with grouping, Table S1: Mass spectrometry identification results of chemical constituents of Atractylodes chinensis (DC.) Koidez., Table S2: List of the characteristic compounds to discriminate AC according to the geographical origin.
