# Supplementary material for: Non-Targeted Metabolomics Combined with Chemometrics by UHPLC–Orbitrap–HRMS and Antioxidant Activity of Atractylodes chinensis (DC.) Koidez. from Eight Origins
Source: Metabolites. 2023 Jul 27;13(8):888. doi: 10.3390/metabo13080888 (PMC10456645; doi:10.3390/metabo13080888)
Supplement: Supplementary file 1 [file metabolites-13-00888-s001.zip › metabolites-2453087-supplementary.pdf]

Supplementary Material

Non-Targeted Metabolomics Combined with Chemometrics by UHPLC–Orbitrap–HRMS and Antioxidant Activity of *Atractylodes chinensis* (DC.) Koidez. from Eight Origins

Xueyan Gao [1,2,3], Danyang Ma [1,2,3], Kaiyuan Li [1,2,3], Tianjiao Xing [1,2,3], Xiwu Liu [4], Lingfeng Peng [1,2,3], Dawei Chen [5,*] and Zhihui Hao [1,2,3,*]

[1] Chinese Veterinary Medicine Innovation Center, College of Veterinary Medicine, China Agricultural University, Beijing 100193, China
[2] Key Biology Laboratory of Chinese Veterinary Medicine, Ministry of Agriculture and Rural Affairs, Beijing 100193, China
[3] National Center of Technology Innovation for Medicinal Function of Food, National Food and Strategic Reserves Administration, Beijing 100193, China
[4] Qingdao Animal Husbandry Workstation, Qingdao 266100, China
[5] NHC Key Laboratory of Food Safety Risk Assessment, Chinese Academy of Medical Science Research Unit (No. 2019RU014), China National Center for Food Safety Risk Assessment, Beijing 100021, China
* Correspondence: chendw@cfsa.net.cn (D.C.); haozhihui@cau.edu.cn (Z.H.)

List of Tables and Figures

Table S1. Mass spectrometry identification results of chemical constituents of *Atractylodes chinensis* (DC.) Koidez.

Table S2. List of the characteristic compounds to discriminate AC according to the geographical origin

Figure S1. The total ion chromatograms of the UHPLC-Orbitrap-HRMS analysis of *Atractylodes chinensis* (DC.) Koidez. samples, with a focus on the (a) negative ion and (b) positive ion.

Figure S2. The chemical structure of 76 compounds identified in this study.

Figure S3. Mass spectrometric fragmentation patterns of (a) Adenosine, (b) L-Phenylalanine, and (c) Scopoletin.

Figure S4. OPLS-DA results of AC from 8 different regions with grouping.

Table S1. Mass spectrometry identification results of chemical constituents of *Atractylodes chinensis* (DC.) Koidez.

No.	Name	Formula	Ionmode	RT [min]	Error [ppm]	MS fragment [*m/z*]	Calculate. MW	MS^2 fragments [m/z]	Classification
1	L-Tyrosine	$C_9H_{11}NO_3$	+	2.041	-2.27	182.08104	181.0735	165.05469, 147.04419, 136.07579, 123.04431, 119.04945, 91.05481	Amino acids
2	Adenosine	$C_{10}H_{13}N_5O_4$	+	2.094	-3.37	268.10333	267.0959	136.06184, 119.03561, 85.0291, 57.0342	Alkaloids
3	2'-Deoxyadenosine	$C_{10}H_{13}N_5O_3$	+	2.165	-2.91	252.1084	251.1011	136.06184, 119.03565, 117.05501, 99.04454, 73.02895	Alkaloids
4	Guanosine	$C_{10}H_{13}N_5O_5$	+	2.193	-3.25	284.09796	283.0908	153.04205, 152.05663, 135.03020, 110.0351, 109.0513	Alkaloids
5	Guanine	$C_5H_5N_5O$	+	2.194	-2.36	152.05635	151.0491	135.03018, 110.03527, 109.05110, 69.0342	Alkaloids
6	2-Furoic acid	$C_5H_4O_3$	+	2.525	-3.07	113.02352	112.0163	97.02898, 95.01339, 85.02930, 73.02917, 69.03438, 67.01877, 57.03429	Organic acids
7	L-Isoleucine	$C_6H_{13}NO_2$	+	2.565	-2.21	132.10175	131.0943	86.09708, 84.08158, 69.07080	Amino acids
8	Chlorogenic acid*	$C_{16}H_{18}O_9$	-	3.162	0.04	353.08789	354.0951	191.05499, 179.03365, 173.04413, 135.04353, 109.02770, 93.03292, 87.00679, 59.01215	Phenylpropanoids
			+	7.681	-2.84	355.10138	354.09408	163.03859, 117.03361, 145.02820	
9	Vanillic acid	$C_8H_8O_4$	+	3.912	-1.64	169.04924	168.042	155.03383, 141.05455, 139.03888, 127.03941, 111.04434, 93.03393, 81.03397, 65.03938	Phenols
10	L-Phenylalanine	$C_9H_{11}NO_2$	+	4.07	-2.3	166.08592	165.0786	131.04950, 120.08102, 103.05472, 93.07050, 91.05493, 79.0549	Amino acids
11	3-p-Coumaroylquinic acid	$C_{16}H_{18}O_8$	-	4.474	1.46	337.09341	338.1007	191.05496, 163.03871, 119.04863, 93.03274	Phenylpropanoids
			+	9.597	-1.87	339.10681	338.09955	215.06976, 148.04713, 147.04376, 119.04919,	
12	Cryptochlorogenic acid	$C_{16}H_{18}O_9$	-	5.131	0.05	353.0878	354.0951	191.05505, 179.03375, 173.04424, 135.04352, 93.03278, 85.02778	Phenylpropanoids
			+	8.198	-2.07	355.10162	354.09408	181.04922, 163.03868, 119.04919, 117.03361	
13	Neochlorogenic acid*	$C_{16}H_{18}O_9$	+	5.846	-2.84	355.10159	354.0941	193.04901, 163.03893, 145.02802, 135.04422, 117.03379, 107.04957	Phenylpropanoids
			-	3.029	0.49	353.0881	354.09525	191.05522, 179.03384, 135.04356, 134.03581	
14	5-O-Feruloylquinic acid	$C_{17}H_{20}O_9$	-	6.015	0.33	367.10342	368.1109	193.04924, 191.05504, 173.04359, 155.03311, 134.03568, 93.03281, 87.00692	Phenylpropanoids

No.	Name	Formula	Ionmode	RT [min]	Error [ppm]	MS fragment [*m/z*]	Calculate. MW	MS^2 fragments [m/z]	Classification
15	Indole-3-acrylic acid	$C_{11}H_9NO_2$	+	6.293	-2.11	188.07022	187.0629	170.0604, 146.06007, 144.08092, 143.07344, 118.06543, 117.07003, 115.05456, 91.05455	Miscellaneous
16	D-Tryptophan	$C_{11}H_{12}N_2O_2$	+	6.294	-2.51	205.09665	204.0894	188.07066, 146.06004, 144.08080, 118.06543, 132.08089, 130.06517, 115.05450, 91.05484	Amino acids
			-	5.032	1.24	203.08176	204.08894	159.09151, 142.06470, 117.05414, 116.04893,	
17	4-p-Coumaroylquinic acid	$C_{16}H_{18}O_8$	-	6.443	1.29	337.09329	338.1006	173.04424, 163.03841, 119.04894, 93.03278, 67.01717	Phenylpropanoids
			+	10.153	-1.78	339.10684	338.09954	215.06981, 197.05927, 169.06445, 147.04376	
18	Phellavin	$C_{26}H_{32}O_{12}$	-	7.473	0.5	535.18213	536.1896	373.12866, 109.02774, 89.02255, 71.01205	Flavonoids
19	4-O-Feruloylquinic acid	$C_{17}H_{20}O_9$	-	7.613	0.29	367.10336	368.1108	193.04948, 173.04424, 134.03571, 117.03310, 93.03280,67.01711, 59.01212	Phenylpropanoids
			+	10.812	-2.14	369.11722	368.11005	177.05415, 149.05942, 145.02815, 117.03344	
20	Caffeic acid	$C_9H_8O_4$	+	7.882	-2.33	181.04918	180.0418	179.03421, 163.03888, 137.05957, 135.04413, 117.03366, 107.04984, 91.05464, 89.03927	Phenols
21	3-O-Feruloylquinic acid	$C_{17}H_{20}O_9$	+	8.31	-1.69	369.11688	368.1101	177.05447, 149.05972, 145.02835, 133.02855, 117.03381	Phenylpropanoids
			-	5.672	0.28	367.10349	368.11083	193.04955, 191.03397, 175.03868, 173.04427, 149.05928	
22	Icariside F2	$C_{18}H_{26}O_{10}$	-	9.624	0.48	401.14536	402.1528	113.02279, 99.00706, 83.01206, 73.02779, 71.01208, 59.01209	Phenols
23	ar-Turmerone	$C_{15}H_{20}O$	+	10.328	-2.9	217.15807	216.1508	189.16357, 175.14807,145.10118, 133.10124, 119.08582, 105.07043, 95.08601, 81.07056	Terpenoids
24	4,6,2',4'-Tetramethoxychalcone 2'-beta-glucoside	$C_{25}H_{30}O_{11}$	-	10.985	1	505.17175	506.1793	487.16052, 475.15967, 343.11899, 89.02265, 59.01197	Flavonoids
25	D-Camphor	$C_{10}H_{16}O$	+	11.452	-2.27	153.127	152.1198	135.11683, 125.05998, 109.10164, 107.08600, 93.03409, 81.07062, 67.05423, 65.03938	Terpenoids
26	Syringaldehyde	$C_9H_{10}O_4$	+	11.628	-2.33	183.06477	182.0575	155.07031, 140.04678, 125.02361, 123.04424, 95.04968, 81.03426, 65.03938, 55.01874	Phenols

No.	Name	Formula	Ionmode	RT [min]	Error [ppm]	MS fragment [*m/z*]	Calculate. MW	MS^2 fragments [m/z]	Classification
27	4-Formylphenyl beta-D-ribopyranoside	$C_{12}H_{14}O_6$	+	11.866	-3.36	255.08545	254.0782	227.09103, 195.06525, 177.05466, 167.07024, 123.04405, 107.0493, 79.05488, 71.04996	Glycosides
28	Icariside D1	$C_{19}H_{28}O_{10}$	-	11.966	0.81	415.16135	416.1686	191.05492, 113.02245, 99.00703, 89.02268, 72.99126	Glycosides
29	Scopoletin	$C_{10}H_8O_4$	+	12.102	-2.58	193.04893	192.0418	178.02605, 165.05510, 150.03107, 149.05988, 137.05975, 133.02850, 122.03655	Phenylpropanoids
30	Abscisic acid	$C_{15}H_{20}O_4$	-	12.227	-0.3	263.12285	264.1361	245.11740, 219.13812, 203.10664, 201.12767, 163.11142, 161.09605, 135.07957	Terpenoids
31	Ferulic acid	$C_{10}H_{10}O_4$	+	12.343	-2.2	195.06487	194.0575	193.04985, 177.05466, 149.05969, 145.02847, 117.03382, 91.05466, 89.03918	Phenylpropanoids
32	Dehydrodiisoeugenol	$C_{20}H_{22}O_4$	+	12.39	-2.67	327.15826	326.1509	295.13275, 175.07626, 163.07530, 137.05972, 133.06473, 131.04929, 122.03649, 105.07018	Lignans
33	(5S,6S)-5-hydroxy-4-methoxy-6-(2-phenylethyl)-5,6-dihydro-2H-pyran-2-one	$C_{14}H_{16}O_4$	+	12.422	-3.3	249.11139	248.104	204.7819, 187.07530, 159.08058, 131.08563, 129.07022,105.07033, 93.07023, 91.05471	Lactones
34	Isofraxidin	$C_{11}H_{10}O_5$	+	12.907	-3.19	223.05937	222.0521	190.02608, 179.03410, 163.03908, 162.03108, 135.04410, 134.03612, 107.04955, 91.05439	Phenylpropanoids
35	Isochlorogenic acid B*	$C_{25}H_{24}O_{12}$	+	14.549	-1.65	517.133	516.1259	499.12405, 337.09216, 319.08051, 163.03889, 135.04414, 117.03378, 107.04945	Phenylpropanoids
			-	11.561	0.31	515.11945	516.12694	353.08810, 191.03384, 179.03369, 173.08041, 135.04355	
36	Isochlorogenic acid A*	$C_{25}H_{24}O_{12}$	+	14.8	-1.35	517.13336	516.1261	449.12115, 337.09009, 319.08017, 163.03889, 135.04411, 117.03378, 89.03913	Phenylpropanoids
			-	10.349	0.46	515.1193	516.12702	353.08786, 191.03386, 179.03375, 173.04428, 135.04352	
37	Isochlorogenic acid C	$C_{25}H_{24}O_{12}$	+	15.942	-1.23	517.1333	516.1261	500.12924, 449.12418, 163.03893, 135.04416, 117.03381, 107.04971	Phenylpropanoids
			-	9.187	0.28	515.11957	516.12692	353.08807, 335.07858, 173.08076	
38	Wikstromol	$C_{20}H_{22}O_7$	+	16.088	-2.42	375.14282	374.1357	357.13229, 345.13348, 325.10831, 161.05962, 137.05969, 107.04948, 91.05465, 73.02914	Lignans
39	2-[(2S)-6-Hydroxy-6-methyl-2-heptanyl]-5-methylphenyl alpha-D-glucopyranoside	$C_{21}H_{34}O_7$	+	16.677	-3.16	399.23642	398.2292	219.17441, 163.11218, 135.08078, 111.08079, 109.10167, 85.02901, 69.03426	Glycosides

No.	Name	Formula	Ionmode	RT [min]	Error [ppm]	MS fragment [*m/z*]	Calculate. MW	MS^2 fragments [m/z]	Classification
40	Pinoresinol 4-O-glucoside	$C_{26}H_{32}O_{11}$	-	16.858	0.17	519.18707	520.1946	357.13440, 342.11038, 122.03571, 121.02775, 83.01205, 71.01198, 59.01201	Lignans
41	Curcolonol	$C_{15}H_{20}O_4$	+	17.116	-3.33	265.14249	264.1353	229.12241, 211.11171, 201.12691, 161.05969, 149.05952, 107.04958, 81.07051	Terpenoids
42	(1S,2S)-1-(4-Hydroxy-3-methoxyphenyl)-2-{4-[(1E)-3-hydroxy-1-propen-1-yl]-2,6-dimethoxyphenoxy}-1,3-propanediol	$C_{21}H_{26}O_8$	+	17.396	-1.8	407.16931	406.162	193.08591, 150.06783, 149.05981, 133.06496, 118.04189, 107.04964, 91.05482, 79.05488	Miscellaneous
43	7-methoxy-2-methyl-2-(4-methylpent-3-enyl)-2H-chromene	$C_{17}H_{22}O_2$	+	17.442	-2.61	259.16852	258.1613	203.10684, 189.09143, 161.05980, 137.05981, 111.08081, 109.10146, 81.07052, 79.05479	Miscellaneous
44	Reynosin	$C_{15}H_{20}O_3$	+	17.571	-4.56	249.14758	248.1401	213.12767, 185.13254, 157.10129, 133.10159, 67.05490	Terpenoids
45	6-Prenylcatechin	$C_{20}H_{22}O_6$	-	17.604	0.4	357.13431	358.1418	339.12311, 150.03050, 108.01985	Flavonoids
46	7-(3,4-dihydroxyphenyl)-5-hydroxy-1-(4-hydroxyphenyl)heptan-3-one	$C_{19}H_{22}O_5$	+	17.614	-3.25	331.15292	330.1457	313.14316, 287.12796, 255.10132, 151.07541, 137.05971, 122.03635, 91.05486	Phenols
47	Carpinontriol B	$C_{19}H_{20}O_6$	+	18.771	-2.53	345.13248	344.1251	299.09189, 269.07941, 239.10619, 223.07516, 198.06816, 137.05914	Phenols
48	(1S,2R)-2-methyl-1,2,3,4-tetrahydronaphthalen-1-ol	$C_{11}H_{14}O$	+	19.478	-3.05	163.11125	162.104	145.10133, 131.04921, 117.07024, 105.07030, 103.05463, 93.07030, 91.05466, 79.05488	Miscellaneous
49	Methyl ferulate	$C_{11}H_{12}O_4$	+	19.854	-3.29	209.08017	208.0729	177.05435, 149.05963, 125.05978, 121,06503, 106.04172, 91.05474, 78.04709, 65.03938	Phenylpropanoids
50	Selina-4(14),7(11)-dien-8-one	$C_{15}H_{22}O$	+	20.744	-3.7	219.17343	218.1663	201.16389, 163.11189, 161.13264, 149.09613, 123.11708, 111.08082, 107.08575, 95.08624	Terpenoids
51	2-[(2'E)-3',7'-dimethyl-2',6'-octadienyl]-4-methoxy-6-methylphenol	$C_{18}H_{26}O_2$	+	21.289	-3.27	275.19962	274.1924	257.18979, 229.19518, 133.10126, 119.08578, 107.08574, 105.07025, 91.05483	Polyacetylene

No.	Name	Formula	Ionmode	RT [min]	Error [ppm]	MS fragment [m/z]	Calculate. MW	MS^2 fragments [m/z]	Classification
52	4-hydroxy-6-[2-(2-methyl-1,2,4a,5,6,7,8,8a-octahydronaphthalen-1-yl)ethyl]oxan-2-one	$C_{18}H_{28}O_3$	+	21.293	-3.11	293.21011	292.2029	275.20056, 257.18918, 147.11691, 109.06512, 107.08605, 81.07047, 79.05496	Lactones
53	Caryophyllene oxide	$C_{15}H_{24}O$	+	21.493	-3.59	221.18929	220.1819	203.17961, 175.14815, 163.14832, 161.13274, 147.11690, 109.10161, 95.08611, 81.07054	Terpenoids
54	7-Demethylsuberosin	$C_{14}H_{14}O_3$	+	22.087	-3.2	231.10097	230.0936	175.03903, 163.03931, 131.04935, 119.04976, 91.05491, 69.07069, 53.00300	Coumarins
55	Pinosylvin	C14H12O2	+	22.103	-2.81	213.09036	212.0831	167.08551, 109.02872, 103.05459, 91.0545, 81.03417	Phenols
56	Limonin	$C_{26}H_{30}O_8$	+	22.179	-1.59	471.20053	470.1933	453.19058, 139.03896, 111.04443, 93.03391	Terpenoids
57	Sedanolide	$C_{12}H_{18}O_2$	+	22.38	-2.56	195.13745	194.1302	177.12744, 149.13307, 135.04459, 107.08617, 93.07049, 81.07054, 67.05494	Phthalides
58	octadecatetraenoic acid	$C_{18}H_{28}O_2$	+	22.383	-3.48	277.21512	276.208	259.20566, 163.11162, 161.13290, 133.10107, 119.08583, 93.07054, 81.07060, 79.05486	Organic acids
59	Arctigenin	$C_{21}H_{24}O_6$	+	22.658	-2.59	373.16339	372.1563	355.15503, 313.14279, 189.09081, 151.07533, 137.05975, 122.03642, 91.05475	Lignans
60	(4E,6E,12E)-tetradecatriene-8,10-diyne-1,3-diol-diacetate	$C_{18}H_{20}O_4$	+	23.341	-2.47	301.14285	300.1354	183.08073, 153.06950, 128.06212, 115.05444, 91.05468, 83.04979, 65.03925, 55.05502	Polyacetylene
61	(9Z,11E,15Z)-13-hydroxy-9,11,15-octadecatrienoic acid	$C_{18}H_{30}O_3$	+	23.516	-3.57	295.22571	294.2184	259.20779, 165.12743, 121.10123, 111.08066, 81.07054, 79.05496, 67.05501	Organic acids
62	Tetraneurin A	$C_{17}H_{22}O_6$	+	23.77	-2.88	323.14819	322.1407	203.10699, 155.07065, 95.04971, 79.05489, 69.03425, 67.05506, 55.01873	Terpenoids
63	Saussureamine B	$C_{20}H_{27}NO_4$	+	24.167	-2.84	346.20035	345.193	231.13791, 213.12729, 163.07541, 116.07098, 79.05499, 70.06595, 69.07061	Terpenoids
64	(6E,12E)-Tetradecadiene-8,10-diyne-1,3-diol	$C_{14}H_{18}O_2$	+	24.232	-3.35	219.13716	218.13	201.12755, 147.08022, 131.08591, 117.07021, 91.05482, 69.03435, 67.05505	Polyacetylene
65	7-phenyl-2-heptene-4,6-diyn-1-ol	$C_{13}H_{10}O$	+	24.63	-2.57	183.07991	182.0727	165.06970, 153.07001, 141.06985, 127.05453, 103.05456, 77.03942	Polyacetylene
66	Atractyloyne	$C_{19}H_{24}O_4$	+	24.774	-2.24	317.1741	316.1668	199.11232, 157.06467, 10106036, 91.05487, 83.04984, 67.05494, 55.05516	Polyacetylene

No.	Name	Formula	Ionmode	RT [min]	Error [ppm]	MS fragment [*m/z*]	Calculate. MW	MS^2 fragments [m/z]	Classification
67	1,7-bis(4-hydroxyphenyl)heptan-3-one	$C_{19}H_{22}O_3$	+	24.779	-2.9	299.16327	298.156	157.06470, 145.06479, 121.06526, 115.05467, 103.05462, 91.05483, 83.04986, 79.05495	Phenols
68	5-Hydroxymethylfurfural	$C_6H_6O_3$	+	24.809	-0.86	127.03877	126.0316	109.02885, 99.04475, 85.02895, 81.03416, 71.04996, 67.01865, 53.00322	Miscellaneous
69	Atractylenolide Ⅲ*	$C_{15}H_{20}O_3$	+	25.01	-4.02	249.14754	248.1403	231.13794, 189.09100, 185.13263, 163.07533, 149.06004, 105.07038, 93.07052, 69.07064	Terpenoids
			-	25.001	-1.73	247.13351	248.14082	203.14348, 187.11194, 147.07993	
70	Furanodienone	$C_{15}H_{18}O_2$	+	25.014	-4.06	231.13687	230.1297	213.12697, 175.07526, 163.07527, 161.05975, 119.08567, 95.08593, 93.07043, 69.07084	Terpenoids
			-	24.889	-2.14	229.12303	230.13019	179.03377, 122, 94,91	
71	4-Methoxycinnamaldehyde	$C_{10}H_{10}O_2$	+	25.015	-2.14	163.07465	162.0677	161.05959, 135.08040, 131.04906, 115.05490, 107.04958, 105.07030, 91.05482, 79.05498	Phenylpropanoids
72	2-(biphenyl-4-yl)acetaldehyde	$C_{14}H_{12}O$	+	25.036	-2.98	197.09546	196.0882	178.07758, 152.06200,103.05472, 91.05478, 65.03915	Miscellaneous
73	8-Epiiridodial glucoside	$C_{16}H_{26}O_7$	+	26.238	-3.41	331.17407	330.1667	127.03918, 97.02892, 85.06548, 69.03432, 57.07081	Terpenoids
74	Atractylenolide II*	$C_{15}H_{20}O_2$	+	26.345	-3.78	233.15263	232.1455	215.14339, 189.14825, 177.09103, 151.07529, 133.06502, 105.07033, 95.08626, 81.07050	Terpenoids
75	Acetylatractylodinol	$C_{15}H_{12}O_3$	+	26.39	-3.27	241.08517	240.0779	213.09074, 199.07545, 198.06767, 169.06483, 171.08006, 147.04347, 93.03371, 81.03414	Miscellaneous
76	Atractylenolide I*	$C_{15}H_{18}O_2$	+	27.147	-2.71	231.13731	230.1301	213.12764, 185.13251, 157.10117, 143.08565, 142.07767, 131.08542, 129.07039, 95.08589	Terpenoids

*Identified by standard substances.

Table S2. List of the characteristic compounds to discriminate AC according to the geographical origin

Model	Marker name	VIP score	p(corr) value	$Log_2(FC)$
M2	Chlorogenic acid	4.13	-0.87	2.15
	Isochlorogenic acid A	1.40	-0.93	2.14
	4,6,2',4'-Tetramethoxychalcone 2'-beta-glucoside	1.35	-0.92	1.95
	Isochlorogenic acid B	1.27	-0.92	1.85
M3	Scopoletin	5.87	-0.97	3.47
	Atractylenolide II	1.88	-0.81	1.79
M4	L-Phenylalanine	4.38	-0.96	2.40
	L-Isoleucine	2.75	-0.99	3.13
	Guanine	1.25	0.84	-7.48
	L-Tyrosine	1.05	-0.93	2.90
M5	Adenosine	3.10	-0.90	1.97

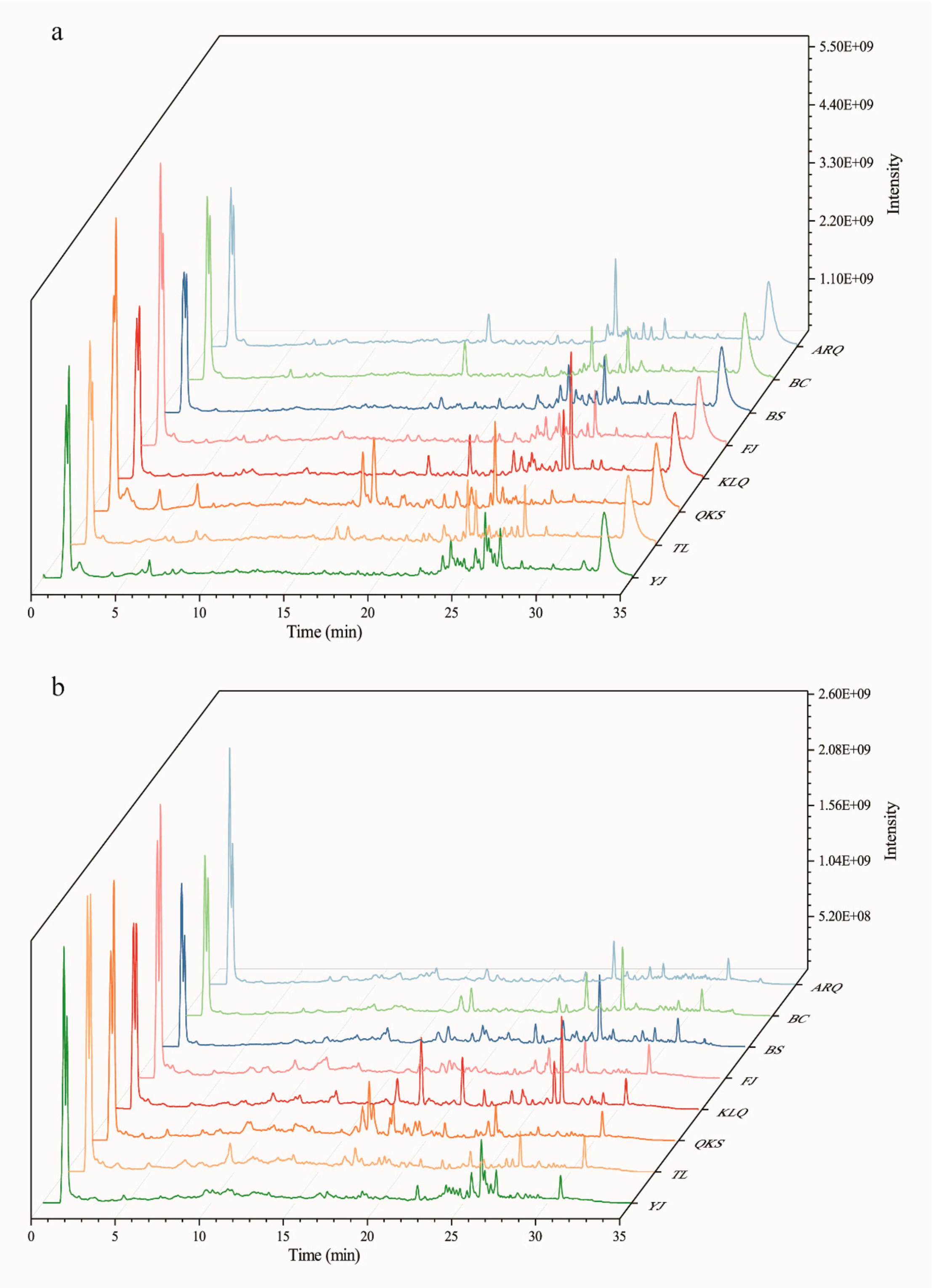

Figure S1. The total ion chromatograms of the UHPLC-Orbitrap-HRMS analysis of *Atractylodes chinensis* (DC.) Koidez. samples, with a focus on the (a) negative ion and (b) positive ion.

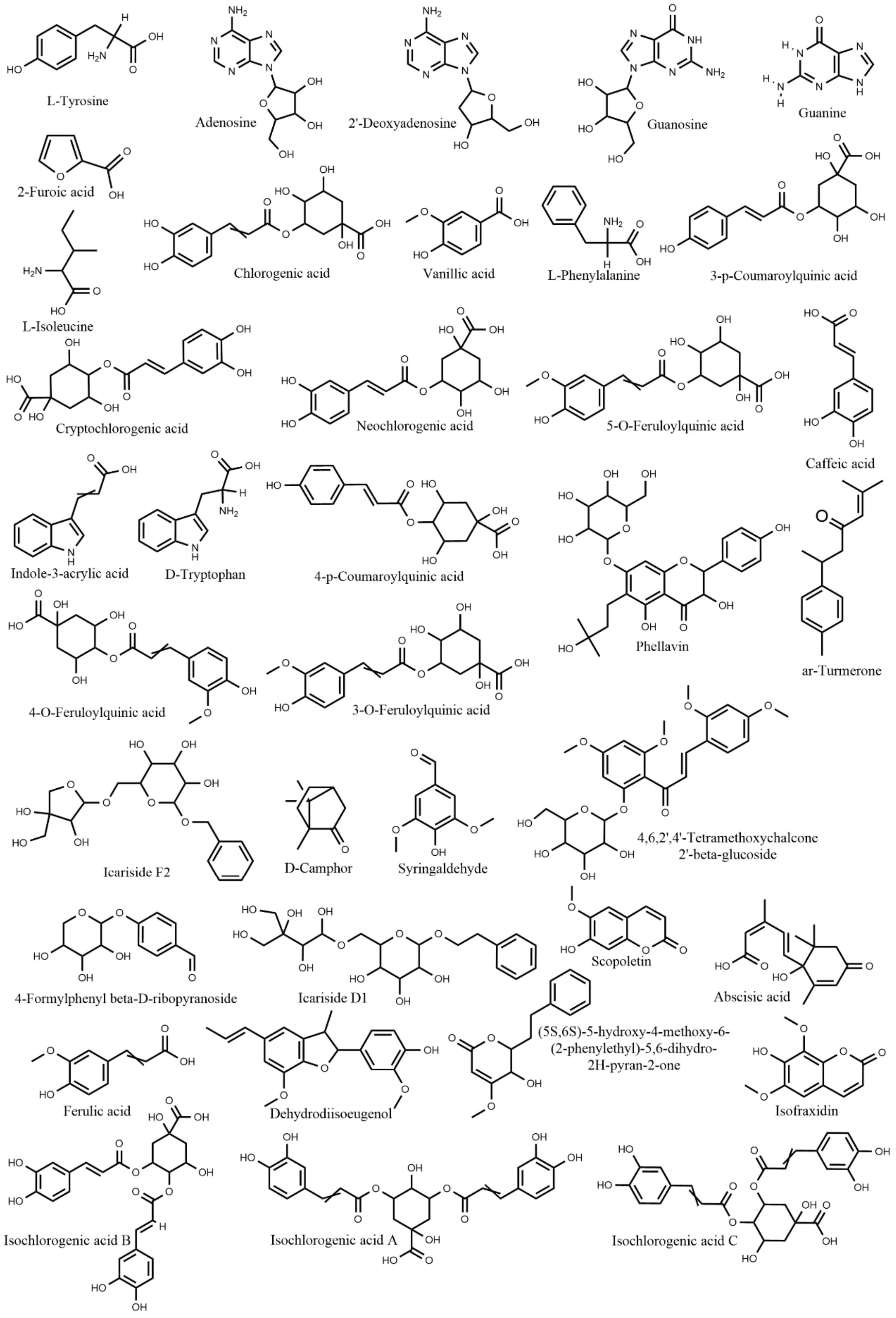
L-Tyrosine
Adenosine
2'-Deoxyadenosine
Guanosine
Guanine
2-Furoic acid
Chlorogenic acid
Vanillic acid
L-Phenylalanine
3-p-Coumaroylquinic acid
L-Isoleucine
Cryptochlorogenic acid
Neochlorogenic acid
5-O-Feruloylquinic acid
Caffeic acid
Indole-3-acrylic acid
D-Tryptophan
4-p-Coumaroylquinic acid
Phellavin
ar-Turmerone
4-O-Feruloylquinic acid
3-O-Feruloylquinic acid
Icariside F2
D-Camphor
Syringaldehyde
4,6,2',4'-Tetramethoxychalcone 2'-beta-glucoside
4-Formylphenyl beta-D-ribopyranoside
Icariside D1
Scopoletin
Abscisic acid
Ferulic acid
Dehydrodiisoeugenol
(5S,6S)-5-hydroxy-4-methoxy-6-(2-phenylethyl)-5,6-dihydro-2H-pyran-2-one
Isofraxidin
Isochlorogenic acid B
Isochlorogenic acid A
Isochlorogenic acid C

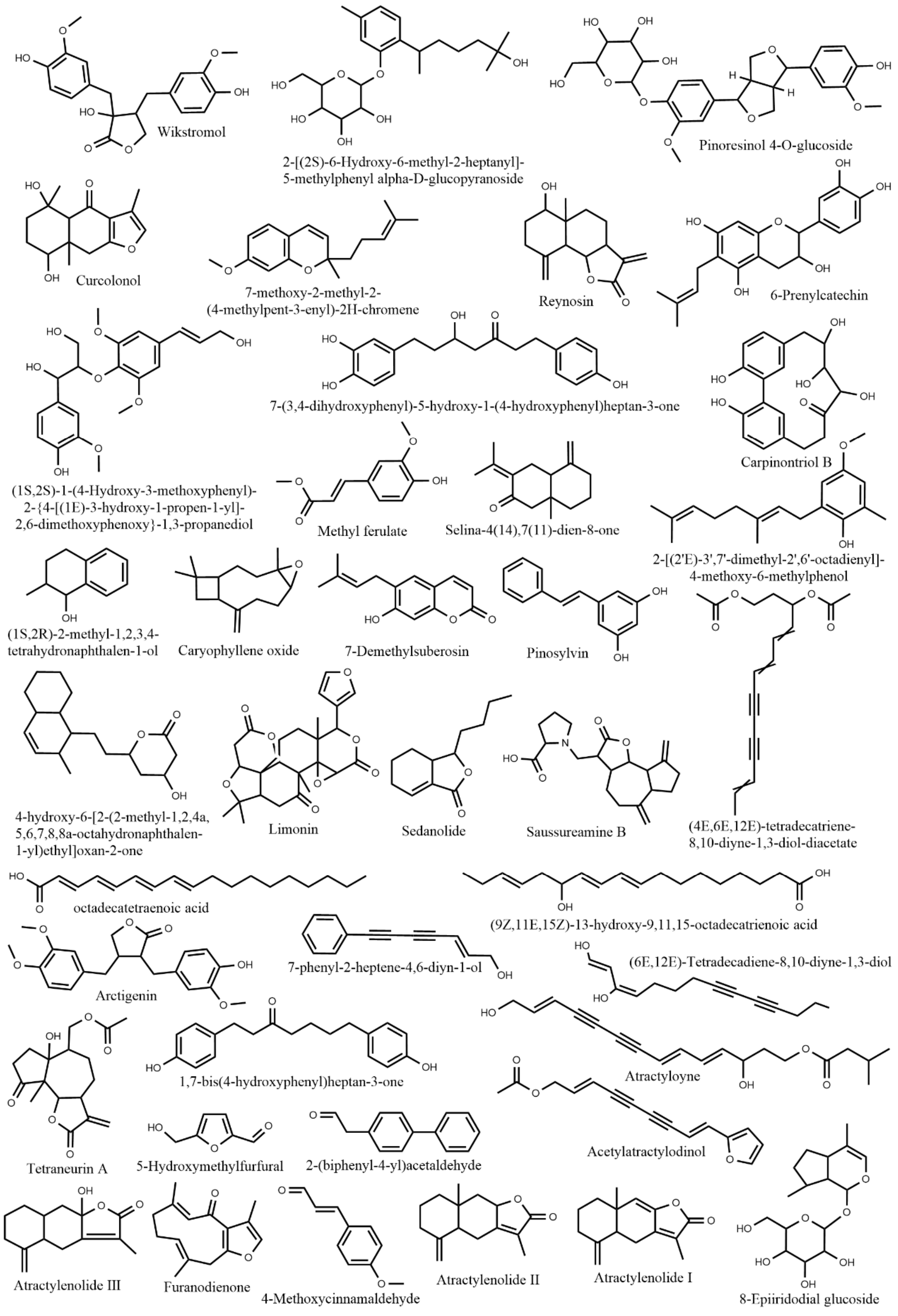

Figure S2. The chemical structure of 76 compounds identified in this study.

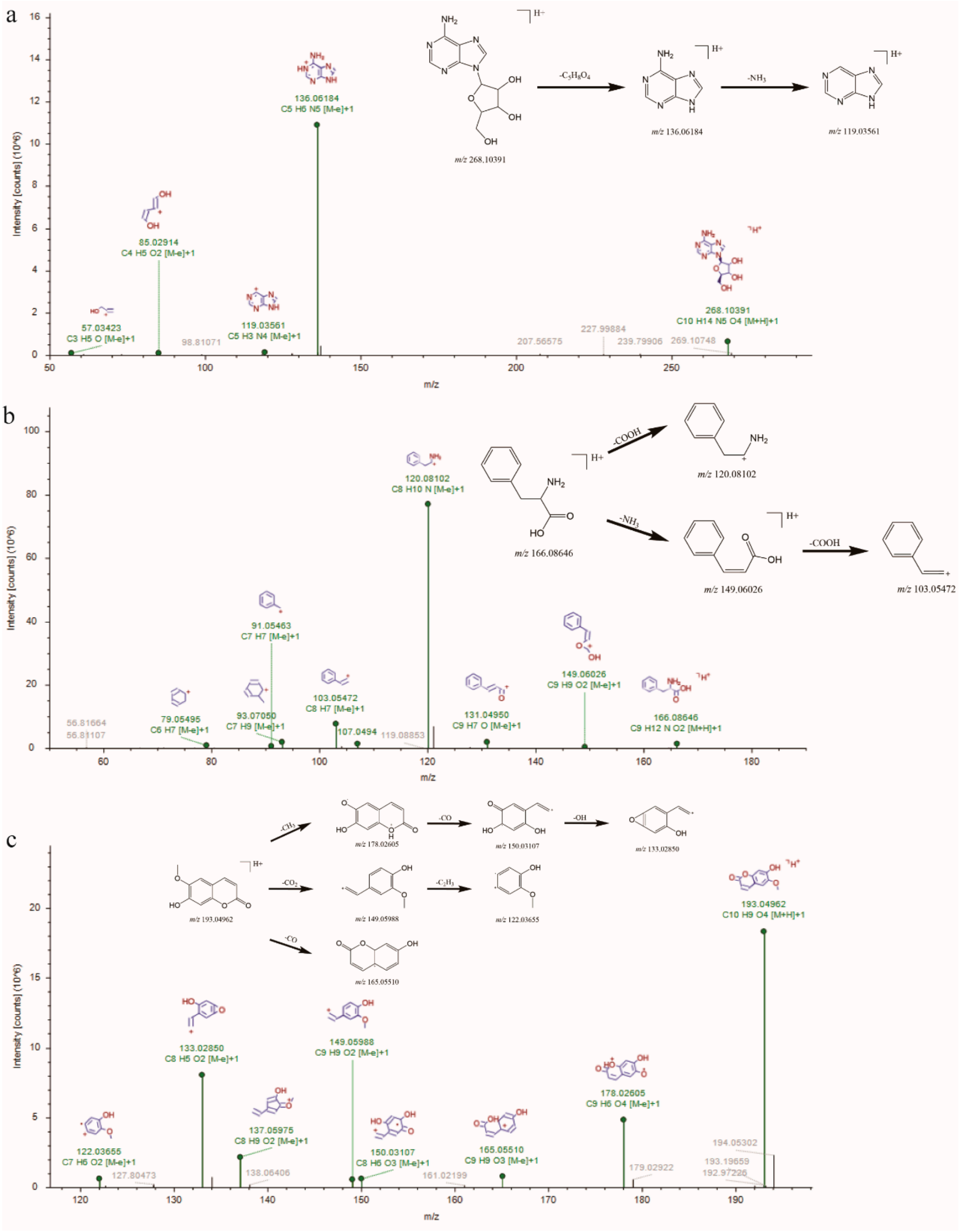

Figure S3. Mass spectrometric fragmentation patterns of (a) Adenosine, (b) L-Phenylalanine, and (c) Scopoletin.

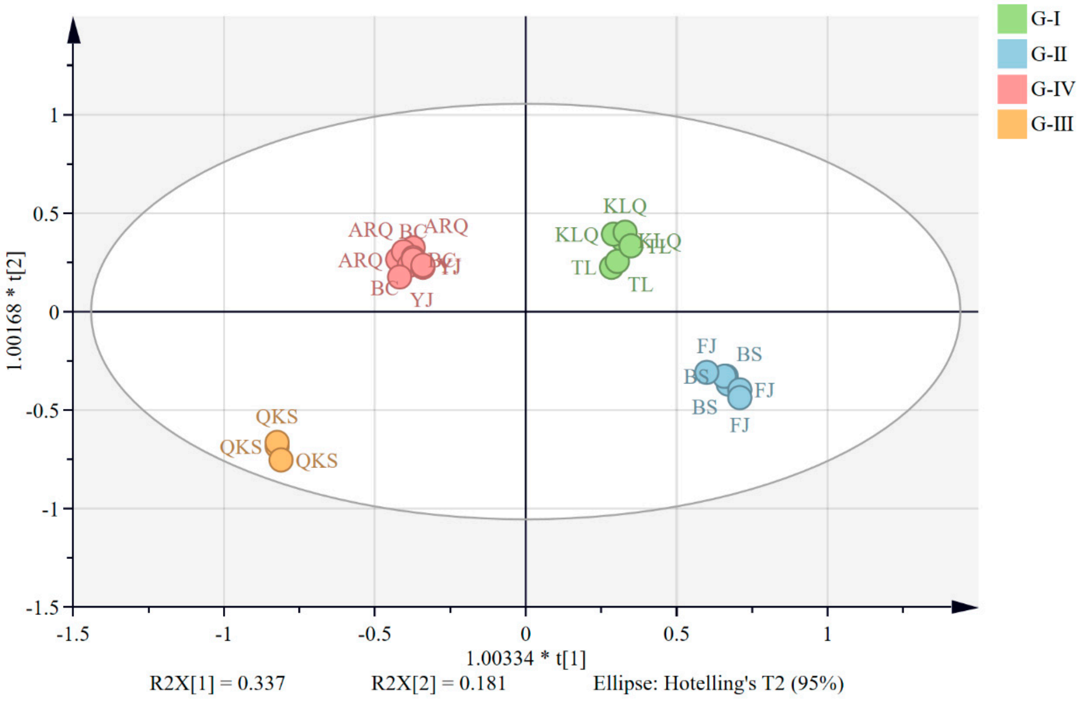

Figure S4. OPLS-DA results of AC from 8 different regions with grouping.
